# The Signaling Pathways Associated With Breast Cancer Bone Metastasis

**DOI:** 10.3389/fonc.2022.855609

**Published:** 2022-03-10

**Authors:** Xuelian Song, Changran Wei, Xiangqi Li

**Affiliations:** ^1^ Department of Breast Surgery, The Second Affiliated Hospital of Shandong First Medical University, Tai’an, China; ^2^ Department of The First Clinical Medical School, Shandong University of Traditional Chinese Medicine, Jinan, China

**Keywords:** breast cancer, bone metastasis, signaling pathway, cytokine, targeted therapy

## Abstract

**Background:**

Breast cancer (BC) is now the leading cause of cancer in women, and bone is the primary site of distant BC metastasis. BC bone metastasis seriously affects the quality of life of patients and increases the mortality rate. However, the mechanism of BC bone metastasis is not fully understood.

**Main Body:**

Paget’s “seed and soil” hypothesis led experts to explore the relationship between surface markers and receptors in breast tumors and various growth factors in bone. The relevant breast tumor markers serve as “seeds”, and the bone microenvironment that is suitable for the survival of the tumor serves as the “soil”. These factors interact to make up an entire system and form feedback pathways that accelerate the production of various cytokines, attracting BC cells to migrate to bone tissue, which worsens the development of BC and seriously affects the prognosis of patients. This process is a vicious cycle. At present, there are seven major signaling pathways involved in BC bone metastasis: the OPG/RANK/RANKL signaling pathway, TGF-β signaling pathway, IGF system, PI3K-AKT-mTOR signaling pathway, Wnt signaling pathway and Hippo signaling pathway. In addition, FGF-FGFR signaling pathway, androgen-AR/LSD1-target gene pathway, Notch signaling pathway, JAK-STAT signaling pathway and CaN/NFATC1 signaling pathway also seem to be associated with BC bone metastasis.

**Conclusion:**

This review focuses on the signaling pathways related to BC bone metastasis and explores the interactions among these pathways, which will lay a solid theoretical foundation for further understanding the mechanism of BC bone metastasis and developing effective targeted therapeutic drugs.

## Introduction

Presently, breast cancer (BC) is the most common malignant tumor and the most important health burden, as well as the most common cause of cancer death among women worldwide. Recently, statistics have shown that the incidence of BC in various countries around the world is increasing at an accelerated rate, and the affected population is becoming younger (1). With the development of imaging technology, surgery and medical treatments, the diagnosis and treatment of BC have improved. The survival rate of BC patients is increased, and the recurrence rate and mortality rate have decreased correspondingly but remain high (2). Studies have shown that metastasis is found in approximately 5% of BC patients at initial diagnosis, and in 20% to 30% of local BC cases, distant metastasis occurs (3). Bone tissue is the most common metastatic site of advanced BC (4) and accounts for approximately 75% of metastasis cases (5), with a 5-year overall survival rate of 22.8% (6) and the highest rate of first recurrence in BC patients. BC bone metastases are mainly osteolytic metastases (7), which cause bone resorption and lead to osteolytic bone lesions. Metastatic bone disease is the result of the interaction between metastatic BC cells and some cells in the bone microenvironment, which indicates that the prognosis is poor and seriously affects the quality of life of these patients. In fact, BC patients with bone metastasis are extremely susceptible to bone-related complications, also known as skeletal-related events (SREs), including bone pain, hypercalcemia, pathological fractures, and spinal cord compression, are commonly used to assess the quality of life and status of patients with BC bone metastases (8). Bone is a site containing many growth factors and cytokines. These factors are released during bone resorption as part of normal bone remodeling or during bone abnormal diseases, such as BC bone metastasis, and affect the proliferation and differentiation of BC cells. Recognized factors on the BC cell surface can spread *via* the blood to any place that is suitable for growth (such as bone) and further development. BC metastasis to bone further promotes the release of bone growth factors and cytokines, accelerating the growth and development of tumor cells, resulting in a vicious cycle. In summary, this review focuses on the signaling pathways related to BC bone metastasis and explores the interactions among these pathways, which will lay a solid theoretical foundation for further understanding the mechanism of BC bone metastasis and developing effective targeted therapeutic drugs.

## Mechanism of Breast Cancer Bone Metastasis

### “Soil and Seed” Hypothesis

Langenbeck once said that “Every cancer cell must be viewed as a living organism capable of development. When plants seed, the seeds are carried in all directions, but they only can survive and grow if they land in the right soil.” In 1882, Fuchs argued that certain organs might be susceptible to secondary cancer. In 1889, Stephen Paget proposed the “seed and soil” hypothesis, which held that tumor cells (seeds) could only grow in fertile soil (bone microenvironment). He argued that in BC, bones suffer in a way that no embolic theory alone could explain (9). Therefore, bone tissue must have a specific microenvironment that supports BC cell growth and development. BC bone metastasis is a complex process involving many kinds of cells and cell growth factors (4). However, it is unclear which cells and cytokines are involved. Therefore, the first thing we need to do is to understand the “soil” and the “seed”.

### “Soil”- Regulatory Cytokines in Bones

**Figure 1:**
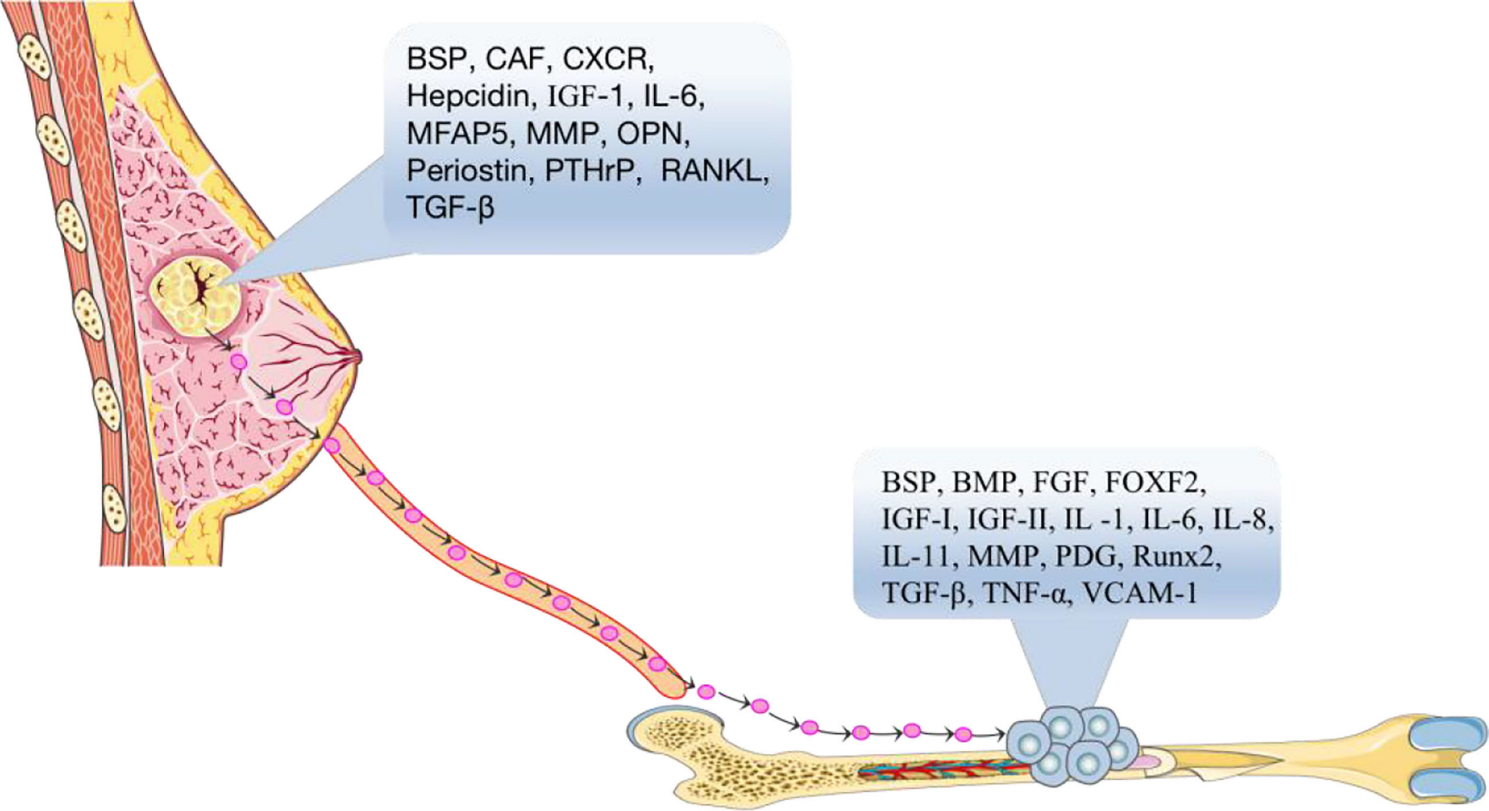
According to the seed and soil hypothesis, the primary breast tumor is the “seed” in the blood that spreads to the suitable growth environment of bone tissue through the actions of cytokines, BSP, CAF, CXCR, hepcidin, IGF-1, IL-6, MMPs, OPN, Periostin, PTHrP, RANKL, TGF-β, etc. BSP, BMP, FGF, FOXF2, IGF-I, IGF-II, IL-1, IL-6, IL-8, IL-11, MMPs, PDG, RUNX2, TGF-β, TNF-α, VCAM-1 and other “soil” factors in the bone microenvironment, which are suitable for tumor cell growth and development, accelerate the process of bone metastasis of tumor cells.

Soil can be defined as a bone microenvironment, containing many cellular growth factors which are the essential factors for growth and metastasis of BC cells (**Figure 1**). Such as transforming growth factor-β (TGF-β) (10), insulin-like growth factor I and II (IGF-I and IGF-II) (11), fibroblast growth factor (FGF) (12), platelet-derived growth factor (PDGF) (13), bone morphogenetic protein (BMP) (14), interleukin-1 (IL-1) (15), interleukin-6 (IL-6) (16), interleukin-8 (IL-8) (17, 18), interleukin-11 (IL-11) (19), tumor necrosis factor-α, (TNF-α) (20), vascular cell adhesion factor-1 (VCAM-1) (21), vascular endothelial growth factor (VEGF) (22), bone sialoprotein (BSP) (23), osteopontin (OPN), and matrix metalloproteinases (MMPs) (24). These cellular growth factors are released by the bone matrix and can bind to receptors on the surface of BC cells and promote the chemotaxis, migration and adhesion of BC cells, allowing BC cells to successfully transfer to the bone.

### “Seeds”- Tumor Markers

Seeds can be defined as the surface markers, which are the identifiable factors, of breast tumor cells (**Figure 1**). It has been reported that BC cells need to interact with receptors in an autocrine manner with the help of the chemokines, cell adhesion factors and growth factors before they can metastasize to bone, and then these cells can further grow and replicate. For example, the surface chemotactic factors on BC cells include CXC chemokine receptor (CXCR) (25), transforming growth factor-β (TGF-β) (10), insulin-like growth factor (IGF) (11), matrix metalloproteinases (MMPs) (26), parathyroid hormone related protein (PTHrP) (27), cancer-associated fibroblasts (CAFs) (28), BSP (29), IL-6 (30), hepcidin (31), periostin protein (32), OPN (29), MFAP5 (33), and RANKL (34, 35).

### “Soil” and “Seeds” Interact in a Vicious Cycle

**Figure 2:**
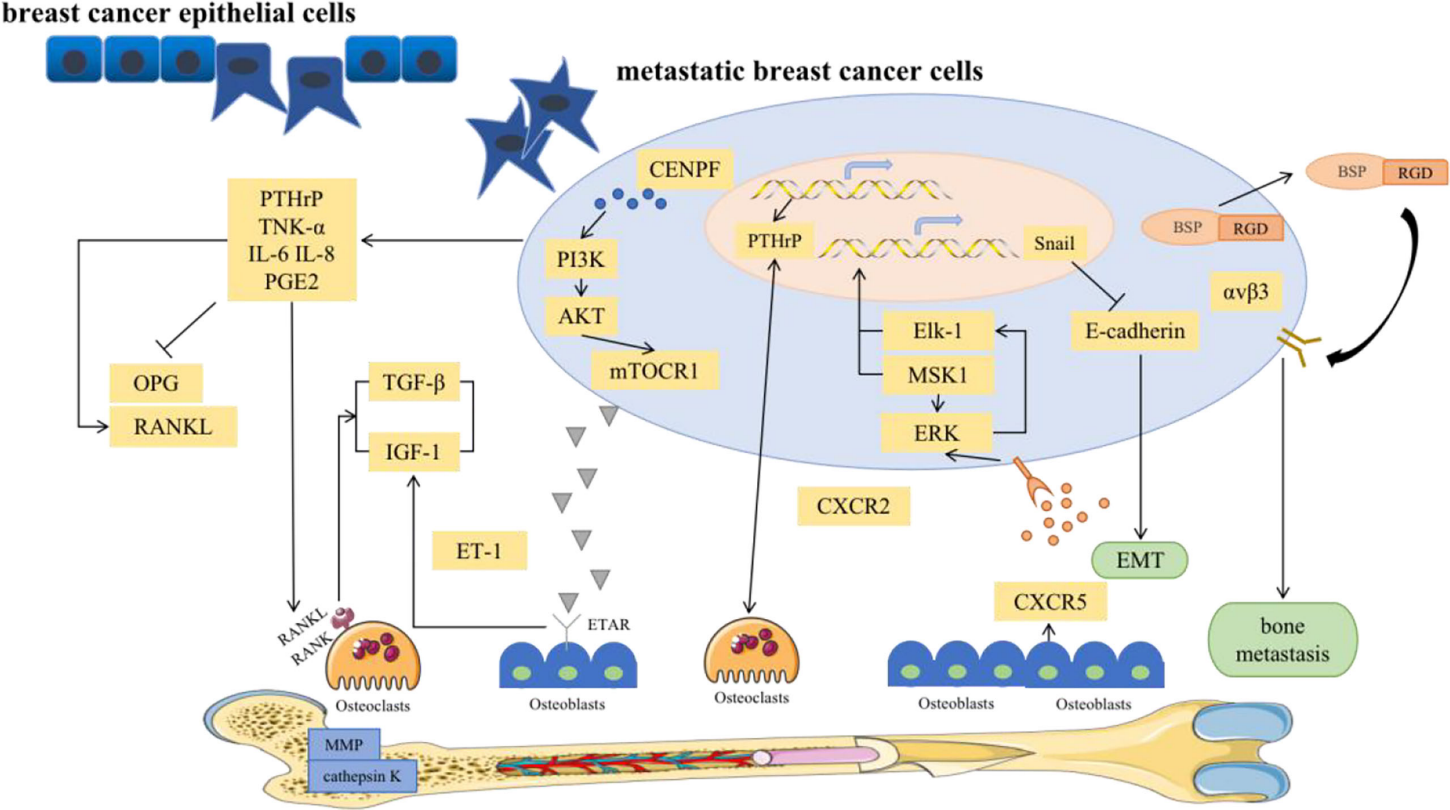
Major signal pathways for bone metastases in breast cancer.

Bone metastases include the spread of cancer cells from the primary tumor *via* the blood to the bone marrow space (4). Notably, BC bone metastases are not directly caused by the destruction of bone but by the upregulation of osteoclasts *via* breast tumor cells, which disrupts the dynamic equilibrium between osteoclasts and osteoblasts. Osteolytic bone metastasis is the main cause of bone destruction. Various pathways have been investigated in the development of bone metastasis (**Figure 2**).

#### OPG/RANK/RANKL Signaling Pathway

The RANKL/RANK/OPG network was originally characterized as a regulator of bone remodeling (36). More and more studies have demonstrated that this network also plays a critical role in osteolysis in metastatic bone diseases, including BC bone metastases (37).

To elaborate, classic OPG, RANK, and RANKL systems provide growth and survival advantages for damaged breast epithelium, which is a prerequisite for BC initiation and a favorable pathway for BC bone metastasis (38, 39). RANK is highly expressed in breast cancer cells. Functionally, it has been shown that RANKL can stimulate the directed migration of mammary epithelial cells toward a source of RANKL (40). RANKL is involved in EMT within breast tumors through up-regulation of Snail. EMT describes a process in which epithelial shaped cells show a transformation into a mesenchymal phenotype. These changes enable cells to invade the surrounding microenvironment leading to cancer progression and an increased stemness of tumors. Stemness and transformation are the main points of action in which RANKL/RANK increases an aggressive behavior of a tumor bone metastasis (39). In addition, high expression of pathways related to T-cell proliferation. RANKL is known to enhance T-cell response and increase dendritic cell survival *via* binding to RANK. RANKL expression on the tumor was associated with downregulation of proliferation and cell cycle-related pathways. Furthermore, RANK expression was associated with activation of immune response and proliferation (41).

In fact, RANKL, its receptor RANK, and the decoy receptor OPG are the key regulators for osteoclast development and the activation of mature osteoclasts. Bone metabolism is thus regulated by a balance between RANKL/RANK signaling and OPG level. RANKL binds to RANK in the bone microenvironment, participates in the maturation of osteoclasts and mediates the activation of the nuclear factor kappa-B (NF-κB) and Jun N-terminal kinase (JNK) signaling pathways, leading to bone resorption. However, excessive absorption can be stopped by osteoprotegerin (42). In solid tumor bone metastasis, the balance between RANKL and osteoprotegerin is affected by many factors, such as PTHrP, vitamin D3 and prostaglandin (39). Among them, PTHrP is the most important osteophilic factor produced by breast tumor cells. PTHrP can reduce the expression of OPG and increase the expression of RANKL in osteoblasts to promote osteoclast formation (39). Thus, PTHrP changes the balance to promote the effects of RANKL. Higher RANKL expression leads to an increased likelihood that osteolysis will be the dominant process. Therefore, BC bone metastasis is more common in the osteolytic environment. In addition, RANKL activates bone resorption by osteoclasts and leads to the release of growth factors (such as TGF-β) from the bone matrix, which in turn stimulates the proliferation of tumor cells (43). Furthermore, metastatic tumor cells can directly secrete RANKL or stimulate osteoblasts to promote the production of RANKL, leading to bone matrix degradation and the release of many bone-derived growth factors and cytokines, which further stimulate the migration of tumor cells to bone (44). The absence of the RANKL/RANK system in breast epithelial cells prevents tumor growth. Importantly, RANKL was observed to be more accurate than conventional markers in the breast cancer patients, and was a good predictor of bone progression. It had been suggested that the RANKL could serve as an accurate marker of bone response in metastatic patients. High RANKL levels may identify patients with a shift in bone homeostasis towards bone resorption who could benefit from bone-targeted treatment aimed at inhibiting osteoclast action (45). Clinical trials have shown that RANKL inhibition significantly delays the occurrence of bone-related events in patients with confirmed bone metastases (46). Therefore, RANKL has become a key factor in the treatment of BC bone metastasis and may provide a unique opportunity for preventing bone metastasis in the future.

#### TGF-β Signaling Pathway

**Figure 3:**
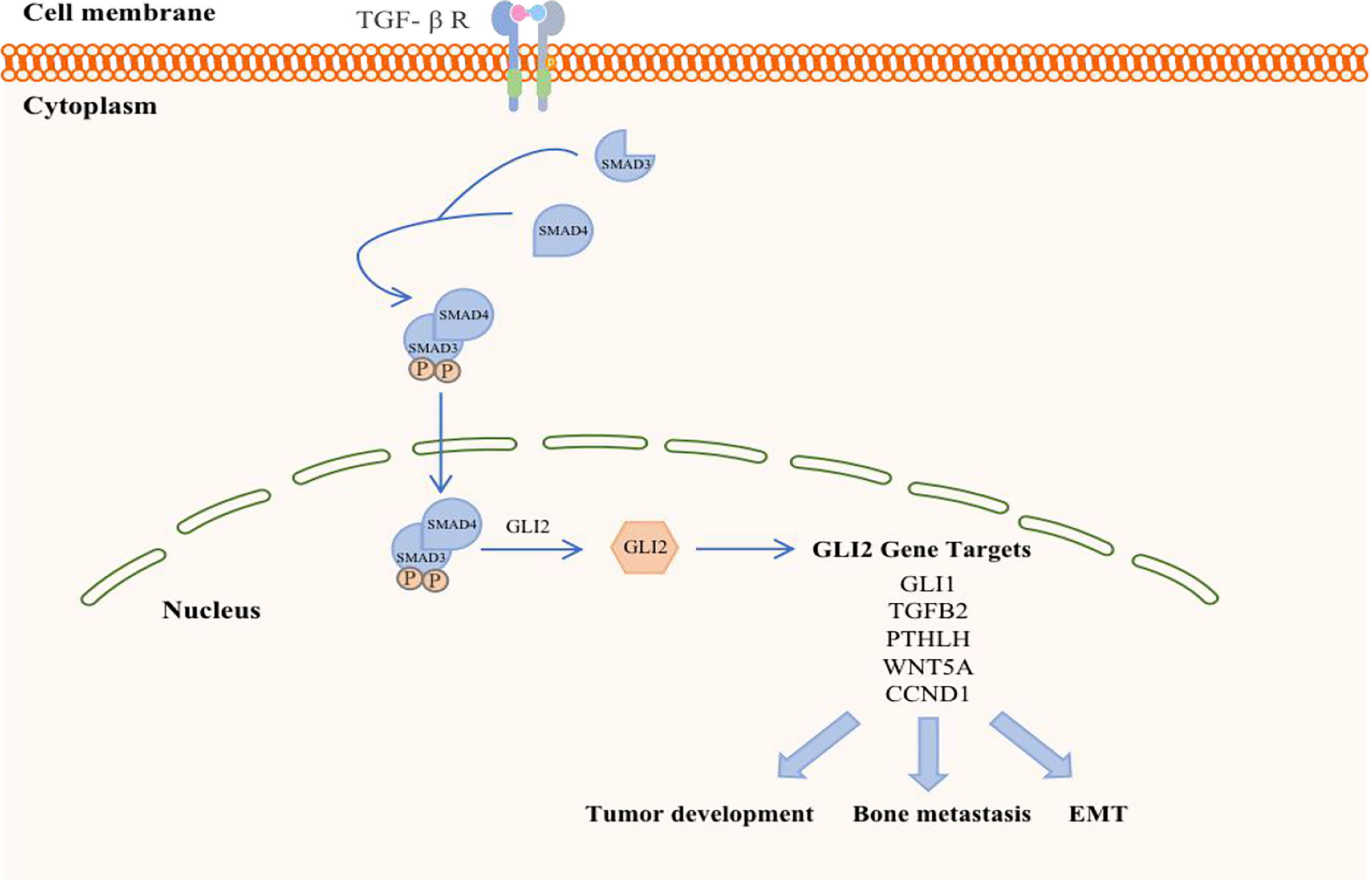
Schematic representation of TGF-β in breast cancer. TGF-β ligand activation of specific cell surface serine–threonine kinase receptors activates the SMAD cascade, resulting in transcriptional activation of the GLI2 gene. GLI2 protein may either regulate target gene expression and exert prooncogenic activities downstream of TGF-β signaling.

The transforming growth factor-β (TGF-β) family members signal *via* membrane-bound, heteromeric, serine-threonine kinase receptor complexes, whose activation by TGF-β ligands leads to phosphorylation of proteins of the SMAD family (**Figure 3**). The latter, in turn, accumulate in the nucleus and act as transcription factors to regulate target-gene expression, acting either directly on SMAD-specific cis-elements on DNA or *via* physical interaction with other transcription factors acting on their cognate DNA recognition sites. Negative control of the cell cycle drives the tumor suppressor functions of TGF-β in normal and premalignant tissues. On the other hand, TGF-β, which is secreted abundantly by tumor cells as well as by the local microenvironment, promotes invasion and metastases of various neoplasms through autocrine and paracrine mechanisms (47, 48). Of note, TGF-β induces epithelial-mesenchymal transition (EMT), whereby epithelial tumor cells acquire an invasive, mesenchymal-like phenotype accompanied by changes in the expression of cell-cell adhesion molecules and secretion of metalloproteinases, leading to metastasis (49). TGF-β is a critical mediator of bone metastasis, whereby complex bidirectional interactions between tumor cells and the bone microenvironment increase bone destruction and establishment of metastases in the bone. In fact, TGF-β signaling blockade by some modalities [i.e., over- expression of either a dominant-negative form of TGF-β receptor type II (50) or SMAD7 (51) in breast cancer cells (52, 53) was shown to be effective in reducing bone metastases.

TGF-β signaling forms a feedback pathway in the occurrence and development of BC, accelerates the progression and metastasis of breast tumors, and participates in the regulation of other pathways to amplify tumor effects (54). First, the TGF-β signaling in BC cells and osteoclasts is activated in the context of BC bone metastasis. BC cells respond to TGF-β stimulation when they reach the bone and promote the maturation of osteoclasts by secreting cytokines such as parathyroid hormone (PTHLH). Mature osteoclasts in turn induce bone digestion, leading to the release of a variety of growth factors, including TGF-β embedded in the bone matrix, which further stimulates breast tumor cells (55). Therefore, the reactivity of TGF-β is a prerequisite for breast tumor cells to initiate osteolytic metastasis. The absence of TGF-β signal transduction in the myeloid system inhibits BC bone metastasis, especially in the development of breast cancer-induced osteolytic bone lesions. Furthermore, bFGF, which is a key molecule in myeloid-specific TGF-β signal transduction, can activate the downstream MAPK-ERK-cFOS pathway by binding to FGFR1 to mediate the development of BC bone damage (10). However, TGF-β signal transduction is not necessary for cellular autonomy in normal physiological conditions (56), and the loss of myeloid-specific TGF-β signaling in bones has other compensatory mechanisms (57).

#### IGF-IGFR Signaling Pathway

In breast tissue, the basic function of IGF is to promote cell proliferation and differentiation through endocrine, paracrine and autocrine mechanisms, and IGF has an antiapoptotic effect. In fact, IGF acts not only on normal cells but also on tumor cells (58). IGF is also widely present in bones. However, the bone microenvironment is conducive for tumor survival because it harbors several growth factors (59), including insulin-like growth factors (IGFs). IGF promotes cancer progression, invasiveness and treatment resistance by activating the IGF receptor (IGFR) and various insulin receptors (11). In the context of bone biology, IGF contributes to the homing, dormancy, colonization and expansion of bone metastases. Preclinical evidence shows that IGF-1 and IGF-1R are the main factors associated with BC bone metastasis. IGF-1, a potent mitogen, activates IGF-1Rβ receptor tyrosine kinase during bone metastasis (60). The activation of IGF-1Rβ triggers downstream signaling including the PI3K/Akt pathway, a critical driver of tumor growth (61)

In primary tumors, a high level of IGF-1 in the environment can promote tumor cell metastasis to bone, suggesting that BC bone metastasis may be IGF-dependent (11). In addition, high expression of IGF-1R is important in malignant cell transformation. Jerome et al. found that IGF-1R could mediate the mitotic functions of normal and malignant breast cells, and high activity and expression of IGF-1R have been associated with BC (62). These results indicate that IGF-1R plays an important role in the pathogenesis of BC. Dunn et al. found that the adhesion, infiltration and metastasis of BC cells are also related to IGF-1R. Metastatic BC cell lines exhibited reduced collagen adhesion by 88% and invasion by 75% after the activation of IGF-1R was blocked by transfection, and when implanted in the breast, distant organ metastasis was also significantly reduced (63). furthermore, the IGF-1Rβ levels are increased in breast cancer metastasis and disruption of IGF-1/IGF-1R signaling inhibited tumorigenesis in preclinical models (64). Therefore, research on drugs related to blocking the activation of IGF-1R will be helpful in blocking BC bone metastasis.

Currently, there are many tumor treatments based on IGF, such as blocking the synthesis and secretion of IGF-1, blocking the binding of IGF-1 to its receptor, downregulating IGF-1, downregulating and antagonizing IGF-1R, and blocking receptor activity (65, 66). These drugs may not only be suitable for the treatment of carcinoma *in situ* but may be effective in patients with BC bone metastases.

#### PI3K-AKT-mTOR Signaling Pathway

**Figure 4:**
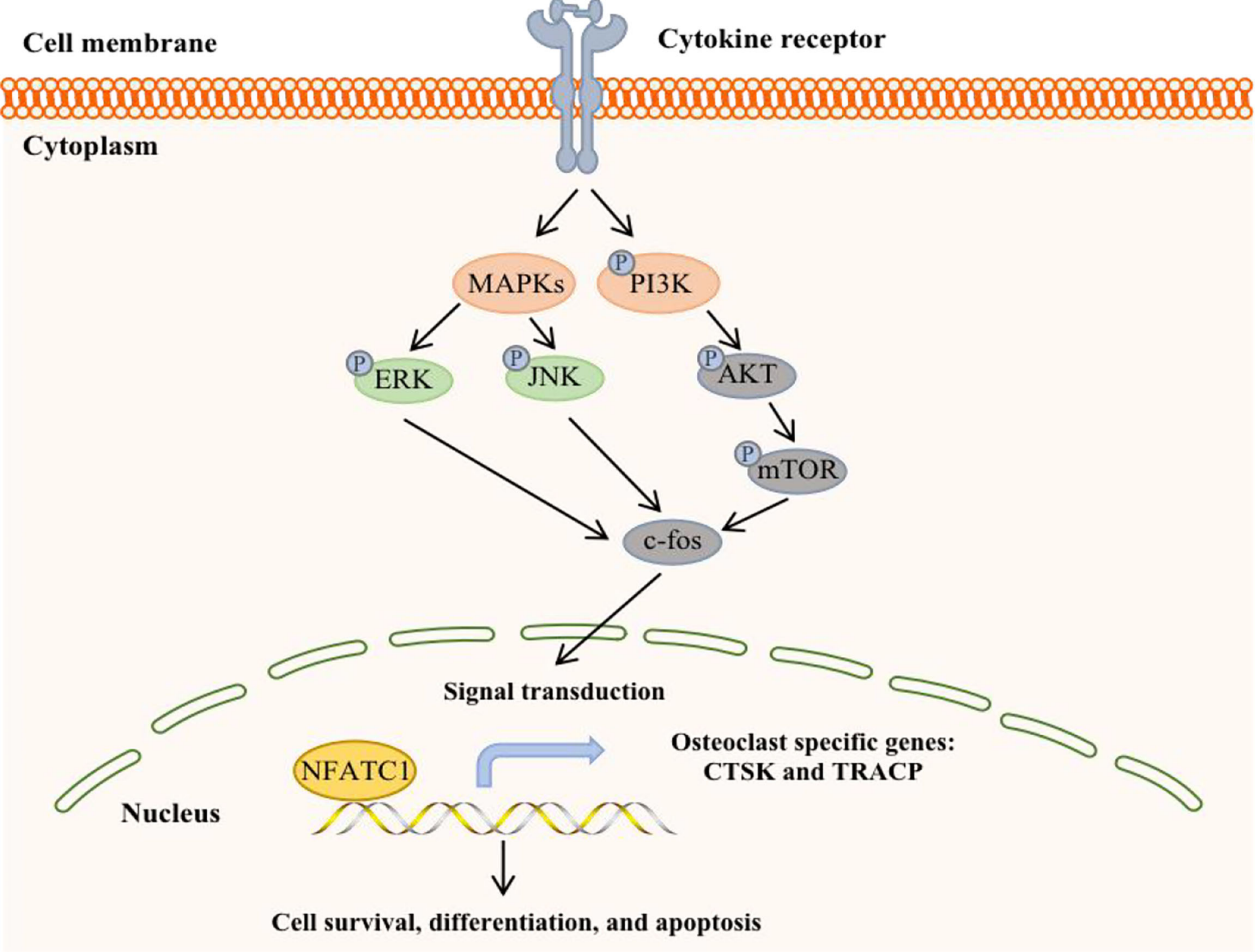
A potential working model in bone metastatic breast cancer.

The phosphoinositide 3-kinase (PI3K)-AKT-mTOR signal transduction pathway is present in a variety of cells in the body and participates in various physiological processes, such as cell metabolism, mitosis, and cell differentiation. This pathway is also one of the most common signaling pathways in a variety of cancers, regulating tumor cell proliferation, apoptosis, invasion and metastasis (67) (**Figure 4**). It is currently known that PI3K/mTOR proteins are highly expressed in human BC, which is associated with the development of breast cancer and osteolysis *in vivo* and *in vitro*. In addition, inhibition of the PI3K-AKT-mTOR signaling pathway can suppress breast cancer growth and breast cancer-induced osteoclast formation (68, 69).

Studies have shown that BC cells invade local lymph nodes *via* the PI3K-AKT-mTOR pathway and promote the expression of MMP-9 (70). mPRα promotes the angiogenesis and metastasis of BC cells by downregulating the connective tissue growth factor-mediated PI3K/AKT/ERK signaling pathway. Furthermore, the level of p-AKT in the spinal cord in BC bone metastasis pain model rats was significantly increased, indicating that the PI3K/AKT signaling pathway was activated. There was a study provides *in vivo* evidence that PI3K/mTOR activity is critical for the metastatic process in a model of bone metastasis. The highly bone metastatic variant of the MDA-MB-231 breast cancer model, 1833, showed PI3K/mTOR activation, high levels of p27pT157 and p27pT198, and p27-dependent motility/invasion *in vitro (* 71). In fact, it has been found that BC bone metastasis could follow the activation of PI3K/protein kinase B (PKB)/AKT/ERK/cAMP response element-binding protein (CREB) signaling pathway, which induces EMT change and MMP2/MMP9 expression. In addition, hypoxia-inducible factor 1α (HIF-1α) and vascular endothelial growth factor (VEGF) are activated through activating the PI3K/AKT/mTOR signaling pathway, which promotes tumor angiogenesis. What is more, colony formation, migration, and invasion of BC cells by downregulating tumor suppressor (p53) and stimulating invasion-associated factor (MMP-9) (72).

In regards to the inhibitors, PKI-402 can induce osteoclast formation and reduce the expression of osteoclast-specific genes in RANKL-induced bone marrow-derived mouse macrophages and reduce the proliferation, migration and invasion of triple-negative breast cancer cell lines, such as MDA-MB-231 and MDA-MB-468 (67). In addition, PKI-402 can also prevent bone destruction caused by BC by inhibiting the PI3K-AKT-mTOR signaling pathway. In addition, GSK690693 is a specific inhibitor of AKT that can inhibit AKT signal transmission, reduce the expression of downstream effectors of p-AKT, and block the downstream signal cascade, thereby exerting biological effects (73). Intrathecal injection of GSK690693 significantly decreased the expression of p-AKT in the spinal cords of rats and inhibited the PI3K/AKT signaling pathway, thereby improving pain symptoms in rats. Furthermore, Asperolide A can inhibit PI3K/AKT/mTOR activation, leading to inhibited proliferation, inhibited migration, inhibited invasion, enhanced apoptosis, and arrest of G2M/S phase cell cycle in breast cancer cells as same as BEZ235 (inhibitor of PI3K/mTOR), and it hindered the phosphorylation of PI3K, AKT, mTOR, JNK, ERK, but not P65 or P38. It is also worth noting that the IGF-1R inhibitor AZD3463 can also inhibit BC bone metastasis by regulating the PI3K-AKT pathway (74). Therefore, there may be a beneficial relationship between the IGF system and the PI3K-AKT-mTOR signaling pathway in BC bone metastasis.

#### WNT Signaling Pathway

**Figure 5:**
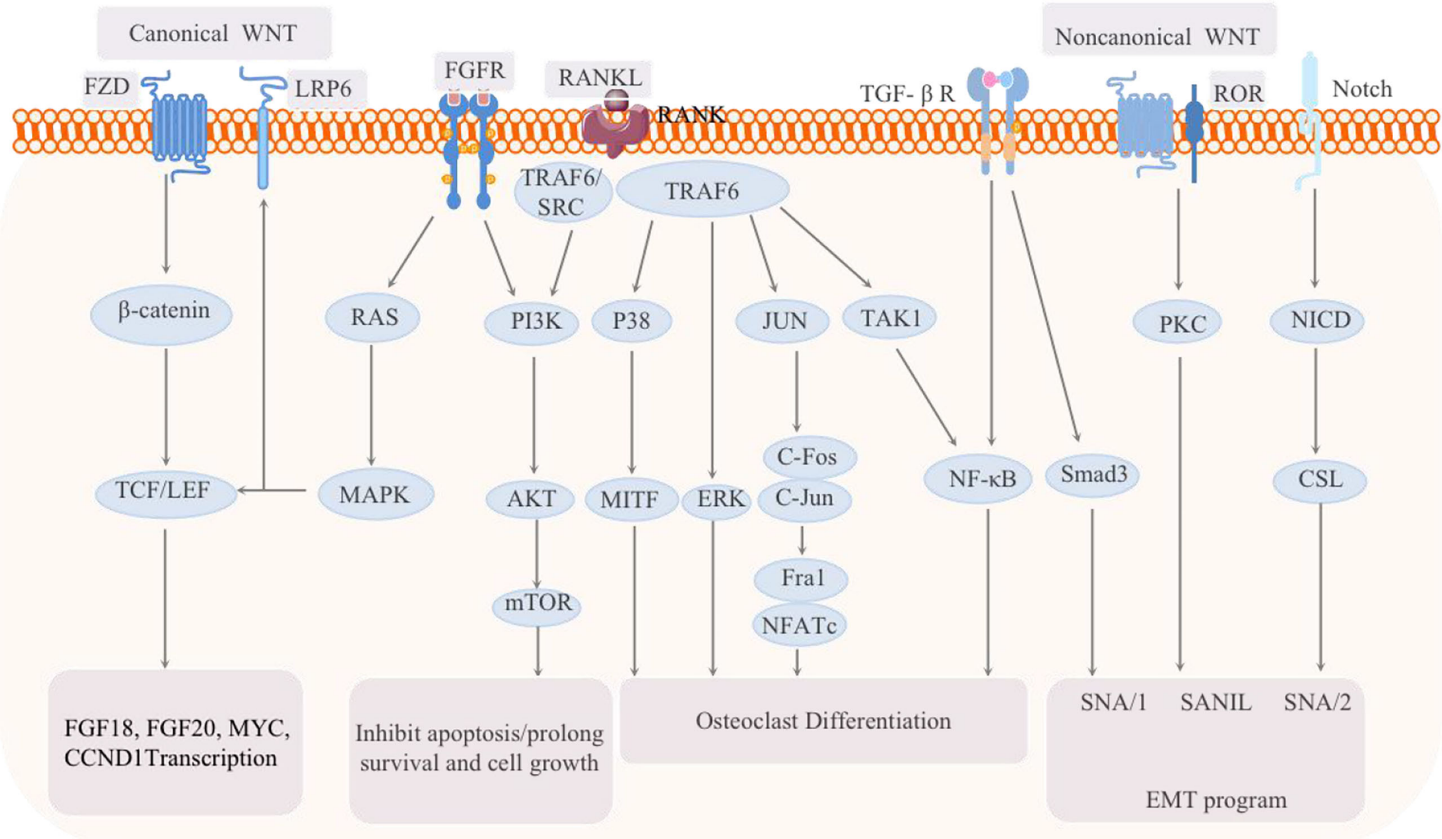
Schematic of the signaling pathways related to breast cancer bone metastasis: the classic WNT pathway and the noncanonical WNT pathway, as well as the FGF-FGFR, RANK-RANKL, TGF, and Notch pathways, are all involved in the regulation of breast cancer bone metastasis.

WNT signaling pathway, consisting of canonical and non-canonical branches, is believed to be responsible for control over various types of stem cells and may act as a niche factor to maintain stem cells in a self-renewing state (**Figure 5**). Moreover, dysregulated Wnt signaling pathway is strongly associated with several diseases including BC (75). Canonical WNT signalling is mediated by β-catenin and activates the TCF/LEF family of transcription factors, while non- canonical WNT/Ca2+ and WNT/planar cell polarity (PCP) pathways are independent of β-catenin. The WNT/Ca2+ pathway activates calcineurin, Ca2+/calmodulin-dependent protein kinase II (CaMKII) and protein kinase C (PKC), further leading to activation of multiple downstream signalling pathways. In fact, Wnt signaling has been shown to be overactivated in both breast cancers induced bone metastases. In particular, WNT signaling act as an important determinant in the cancer induced bone lesions, especially in controlling osteoblastic effect within tumor-harboring bone environment (76, 77).

*In vivo* selection of metastatic cells from independent human breast cancer cell lines yields derivatives that harbor a hyperactive WNT signaling, in association with an enhanced ability to infiltrate and colonize the bones. Two WNT target genes, the transcription factors HOXB-9 and LEF-1, were identified as mediators of chemotactic invasion and colony outgrowth. These findings indicate activation of WNT signaling as a determinant of metastasis to bone during cancer progression. In fact, the overexpression of miR-574-5p seems to be associated with cancer bone metastasis involving aberrant WNT activation. The miR-574-5p, which functions to suppress Qki6/7, was reported to be upregulated in cancer samples that metastasized to bone when in comparison with the non-metastatic samples.

In addition, bone marrow-derived IL-1β stimulates the growth of BC cells in bone by inducing NF-κB and CREB signaling, leading to autocrine Wnt signaling and CSC colony formation (78). Some studies have shown that inhibiting this effect can prevent CSCs from forming colonies in the bone environment and prevent bone metastasis (78). Therefore, targeting IL-1β-NF-κB/CREB-Wnt signal transduction can be used as a therapy to prevent BC bone metastasis. It is currently known that IL-1β is a key cytokine produced by immune cells and nonimmune cells in human bone marrow that can enhance the ability of BC cells to form colonies (79, 80). Recently, it has been shown that tumor-derived Wnt can also promote the secretion of IL-1β by macrophages, which can accelerate the progression of BC bone metastasis; therefore, inhibiting this signal transduction pathway will delay disease progression. IL-1β-NF-κB/CREB Wnt signaling is a new way to promote BC cell colonization in the bone microenvironment. Drugs targeting this pathway can prevent not only bone metastasis *in vivo* but also colony formation of mammary stem cells *in vitro (* 78).

#### Hippo Signaling Pathway

The Hippo signaling pathway plays an important role in the occurrence, development and metastasis of BC. The core component of the Hippo signaling pathway is the transcriptional coactivator protein TAZ, and studies have shown that TAZ expression and activity was upregulated in highly metastatic breast cancer (81). Abelson tyrosine protein kinase (ABL) enhances the expression of TAZ and STAT5, which together promote BC bone metastasis (82). Similar to TAZ, YAP can also act as an oncogene to promote the occurrence and progression of BC *in vivo* and *in vitro*. YAP mainly promotes the growth, progression and metastasis of BC cells by interacting with transcription factors such as TEAD.

MST1/2 and LATS1/2, which are upstream signals of TAZ/YAP, are essential for the regulation of TAZ/YAP (83). The phosphorylation of Tyr1307 by HER3 can methylate the Lys59 site of MST1, which leads to the activation of YAP and TAZ in tumor cells and ultimately promotes bone metastasis (84). Bartucci et al. found that the nuclear expression of TAZ in bone metastases was significantly higher than that in primary tumors (85). It is believed that TAZ and YAP function as oncogenes in BC. However, notably, role of YAP as an oncogene is still controversial. Some researchers have pointed out that YAP can also act as a tumor suppressor gene and inhibit the development of BC. The mechanism remains to be studied in the future.

In addition, the hypoxic microenvironment in the bone marrow is conducive to tumor infiltration, which may regulate BC bone metastasis *via* the Hippo signaling pathway. HIF-1α is considered to be an indicator of hypoxia, and there is evidence that the transactivation of HIF-1 is regulated by the interaction between E-cadherin and Hippo pathway factors (86). Bendinelli et al. reported that HIF-1α interacts with TAZ and stimulates BC bone metastasis in a hypoxic microenvironment (87, 88). Hypoxia enhances the colocalization of TAZ and HIF-1α in the nuclei of human 1833 cells and interferes with the DNA binding activity of the HIF-1-dimer complex. In addition, oxidative stress/COX-2 may be a molecular connection between hypoxia stimulation, the Hippo pathway and the transcriptional regulator Snail. Blocking Cox-2 can downregulate the expression of HIF-1α and Snail in the nuclei of hypoxic 1833 cells and phosphorylate TAZ by interacting with LATS. The localization of LATS in the nucleus can promote the translocation of TAZ in the cytoplasm, mediate the phosphorylation and degradation of TAZ, and inhibit the entry of TAZ into the nucleus, modulating the transcriptional coactivation of TAZ and preventing the occurrence of tumors (88).

Therefore, YAP/TAZ inhibitors and COX-2 inhibitors can be studied in drug research and development and may serve as solutions to prevent tumor progression and reverse the tumor microenvironment to break the vicious cycle.

#### Others

In fact, there are some other pathways associated with breast cancer bone metastasis. For example, bFGF is known to critically regulate self-renewal and proliferation of bone marrow derived MSCs. Therefore, activation of the bFGF pathway in breast cancer cells-bone marrow crosstalk can be speculated to foster a tumorigenic priming of the niche to harbor and support invading tumor cells (89, 90). In addition, some literature notes that there is a significant positive correlation between serum androgen levels and breast cancer risk (91). The androgen-AR/LSD1-target gene pathway seems to also be related to BC bone metastasis. Androgen can activate the estrogen receptor alpha (ERα, ESR1) signaling pathway in BC (92). However, some experts believe that AR is not an oncogene and may a tumor suppressor in some cases (93, 94), and further investigations are needed. The Notch pathway is also involved in BC bone metastasis, and it has been shown to have antagonistic or synergistic effects with the Ras pathway in different environments (95).

## Discussion and Conclusion

Distant metastasis of breast cancer is a complex pathological process that requires multiple steps and is controlled by multiple genes and signaling pathways, including the stages of adhesion, degradation, and migration (96). First, breast tumor cells have reduced adhesion and detach from the original site, and their surface receptors specifically bind to certain components of the basement membrane and extracellular matrix. Second, tumor cells secrete matrix metalloproteinases (MMPs) to degrade the protein components of the ECM, causing the ECM to lose its blocking effect. Eventually, the tumor cells spread from the original site, invade and enter lymphatic and blood vessels *via* defective areas in the ECM and metastasize to the distant site (29). As a nearby organ, bone tissue contains a large number of cytokines and provides conditions and an environment that supports for BC metastasis.

This article summarizes the current pathways related to BC bone metastasis, and the interactions are shown in **Figure 5**. It has been noted that the downstream p38, JNK, NF-κB, ERK and AKT pathways can all be activated during the activation and survival of osteoclasts *via* by the RANKL/RANK pathway (97). In BC T47D cells, RANKL activates the AKT and ERK1/2 pathways. The comprehensive signaling network composed of TGF-β, FGF, NF-κB, WNT, PI3K and JAK-STAT is also essential for the progression of BC bone metastasis. Key bone transfer factors, such as vascular cell adhesion molecule 1 (VCAM1), RANKL, PTHrP, and other important mediators of these pathways, have further confirmed this signaling network (97). It is worth noting that the RANKL/RANK system is an important molecular link between progesterone and epithelial carcinogenesis (35). Therefore, inhibition of this system can also be used to prevent and/or treat hormone-related BC. However, a new question must be considered. Is BC with progesterone receptor positivity more prone to bone metastasis than other subtypes?

In addition, a basic fibroblast growth factor (bFGF)-mediated, synergistic increase in proliferation of breast cancer cells and mesenchymal stromal cells (MSCs) in co-culture. The stromal induction was associated with elevated phosphoinositide-3 kinase (PI3K) signaling in the stroma, which coupled with elevated bFGF levels resulted in increased migration of breast cancer cells towards the MSCs. The perturbed cytokine profile in the stroma led to reduction in the osteogenic differentiation of MSCs *via* downregulation of platelet-derived growth factor-BB (PDGF-BB) (98). FGF and NF-κB are both important inducers of EMT and metastasis, and the NF-κB and TGF-β signaling pathways synergistically enhance EMT and cancer metastasis (12). Wnt signaling is one of the most important ways that induces EMT and breast stem cells (79). In addition, the Wnt and TGF-β signaling pathways may also induce a mutually reinforcing autocrine signaling network, which is essential for the sustained expression of EMT-related transcription factors and CSCs. The FGF15/19-FGFR4 signaling pathway can activate Wnt/GSK-3β/β-catenin (99, 100) and MST1/2 (101, 102). FGFs induce the dimerization, activation and tyrosine phosphorylation of FGFRs and the subsequent phosphorylation of FGFR substrate 2α (FRS2α) and phospholipase Cγ (PLCγ), thereby activating the RAS-ERK and PI3K-AKT-IP3-Ca^2+^, diacylglycerol (DAG)-protein kinase C (PKC) signaling pathways (103). Among them, FGF signal transduction to the RAS-MAPK branch and the typical Wnt signaling cascade mutually regulate transcription; FGF signal transduction to the PI3K-AKT, Hedgehog, Notch, TGF-β and noncanonical WNT signaling cascades regulates EMT and invasion (104). Therefore, there is crosstalk between the FGF-FGFR pathway and the WNT and Hippo signaling pathways. The PI3K signaling pathway also plays an important role in inducing EMT and metastasis (105).

Interesting, it has been found that applied mechanical tension can dramatically alter gene expression in breast cancer cells, leading to decreased proliferation, increased resistance to chemotherapeutic treatment and enhanced adhesion to inflamed endothelial cells and collagen I under fluidic shear stress. A mechanistic analysis of the pathways involved in these effects supported a complex signaling network that included Abl1, Lck, Jak2 and PI3K to regulate pro-survival signaling and enhancement of adhesion under flow. Studies using mouse xenograft models demonstrated reduced proliferation of breast cancer cells with orthotopic implantation and increased metastasis to the bones when the cancer cells were treated with mechanical load (75). Furthermore, hypoxia and hypoxia-inducible factors (HIFs) also play important roles in BC bone metastasis. HIF can directly regulate genes. In addition, some genes are directly regulated by hypoxia, such as TFF3, EGLN1, SNAI1, MMP9, TGFB3, SLC2A3 and CTGF (87). Therefore, hypoxia and HIFs may be important factors that regulate the EMT status of primary and secondary tumors.

In short, signaling pathways interact with each other to promote the development of BC bone metastasis. The secretion of various growth factors creates a vicious cycle, amplifying the signals associated with BC bone metastasis and promoting “seed” germination in the “soil”. It is not enough to understand the soil and seeds alone, and we should also increase efforts to thoroughly understand the various signaling pathways and their potential relationships. This understanding can truly prevent BC bone metastasis and lay a solid foundation for future treatment to relieve the pain and prolong the life of BC patients with bone metastases.

## Author Contributions

XS wrote this manuscript. XS and CW drew the pictures in this paper. In addition, XS, CW, and XL revised the manuscript. All authors contributed to the article and approved the submitted version.

## Funding

The present study was supported by the National Natural Science Foundation of China (grant no. 81473687), the Academic Promotion Program of Shandong First Medical University (grant no. 2019QL017), the Natural Science Foundation of Shandong Province (grant no. ZR2020MH357, ZR2020MH312), Tai’an Science and Technology Innovation Development Project (grant no.2020NS092).

## Conflict of Interest

The authors declare that the research was conducted in the absence of any commercial or financial relationships that could be construed as a potential conflict of interest.

## Publisher’s Note

All claims expressed in this article are solely those of the authors and do not necessarily represent those of their affiliated organizations, or those of the publisher, the editors and the reviewers. Any product that may be evaluated in this article, or claim that may be made by its manufacturer, is not guaranteed or endorsed by the publisher.
